# When comedones mislead: a dermoscopic and histological clue to follicular dowling-degos disease^؆^

**DOI:** 10.1016/j.abd.2025.501256

**Published:** 2025-12-24

**Authors:** Sonika Garg, Anuja Yadav, Gajanand M. Antakanavar, Meeta Singh, Zoya Hasan

**Affiliations:** a Department of Dermatology and Venereology, Faculty of Medical Sciences, Maulana Azad Medical College, University of Delhi, Delhi, India; b Department of Pathology, Faculty of Medical Sciences, Maulana Azad Medical College, University of Delhi, Delhi, India

Dear Editor,

Dowling-Degos Disease (DDD) is a rare, inherited skin condition characterized by a distinctive pattern of dark, lacy (reticulate) pigmentation, particularly in flexural sites. It typically presents in females during the third or fourth decade of life.^1^

A 26-year-old female presented with multiple asymptomatic dark lesions over the face, axillae, groin, inframammary folds, buttocks, and extremities since childhood. The lesions initially appeared in the axillae and gradually progressed to involve other body sites. A positive family history was noted, as similar lesions were observed in her mother. There was no history of itching, photosensitivity, or any systemic complaints.

On examination, numerous folliculocentric brown-to-black macules and comedone-like papules were widely distributed over the body with a predilection for flexural involvement (Fig. 1A‒C). Multiple hyperpigmented atrophic macules and follicular pits were also present over the face (Fig. 1D). Nails, mucosae, palms, and soles were spared. No typical non-follicular, reticulate flexural hyperpigmentation was present.

**Fig. 1:**
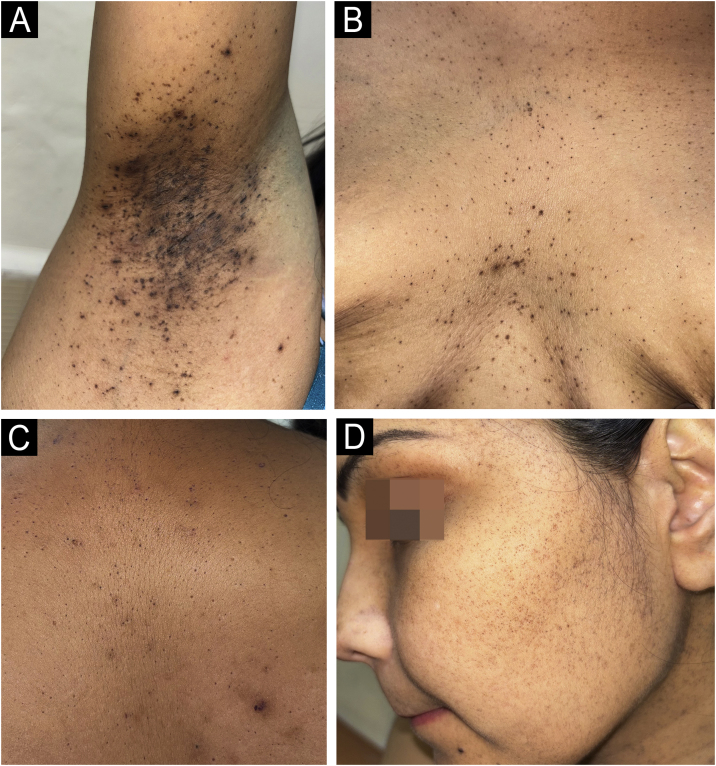
Clinical photographs showing multiple follicular hyperpigmented macules and comedo-like papules distributed over the axillae (A), intermammary region (B), and upper trunk (C), Facial involvement with scattered atrophic macules and follicular pits (D).

Dermatoscopy (polarized mode, 10× magnification) over the upper back revealed folliculocentric, irregular star-shaped, and Chinese letter-like brown pigmentation, multiple discrete dark brown follicular plugs with a surrounding faint reticulate pigment network (Fig. 2A‒B). Based on clinical and dermoscopic findings, a provisional diagnosis of follicular Dowling-Degos Disease (DDD) was considered.

**Fig. 2:**
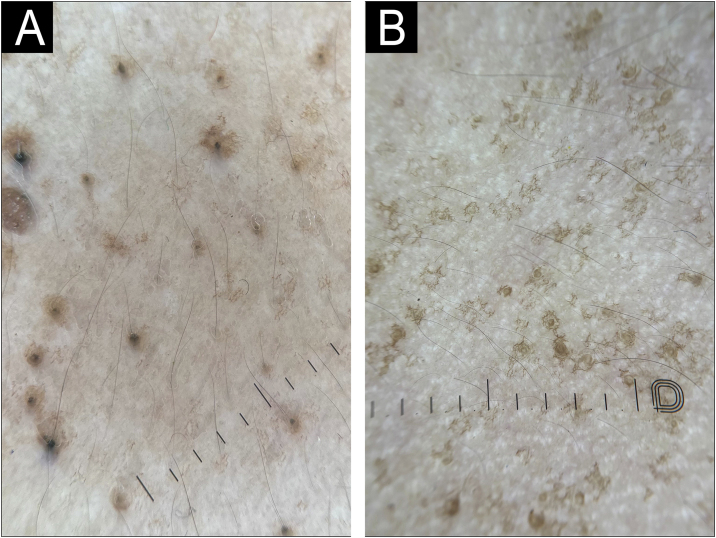
Dermoscopy (polarized, 10× magnification) demonstrating central dark brown follicular plugs surrounded by irregular star-shaped and Chinese letter-like brown pigmentation (A). Dermoscopy (polarized, 10× magnification) demonstrating Chinese letter-like pigmentation centered around a follicular opening (B).

Histopathologic examination from a buttock lesion showed characteristic antler-like elongation of rete ridges, basal layer hyperpigmentation, and follicular plugging with preservation of the suprabasal layer (Fig. 3A‒B). No dyskeratosis, corps ronds, or grains were noted.

**Fig. 3:**
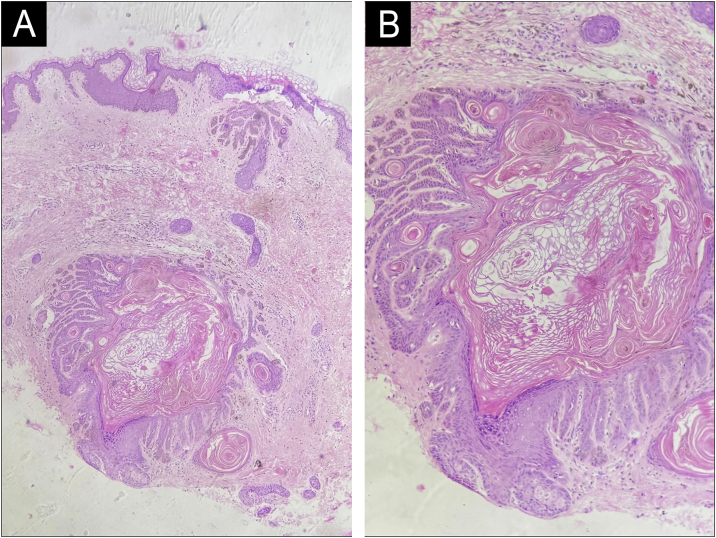
Histopathological image showing elongated, branching (“antler-like”) rete ridges, basal hyperpigmentation, and follicular plugging, with sparing of the interfollicular epidermis (A ‒ Hemaxitolyn & eosin, 40×), (B ‒ Hemaxitolyn & eosin, 100×).

Dowling-Degos Disease (DDD) is a rare pigmentary disorder caused by mutations in the KRT5 gene, inherited in an autosomal dominant pattern or occurring sporadically. It typically presents in females during the third or fourth decade of life.^1^ The KRT5 gene, located on chromosome 12, is essential for maintaining keratinocyte structure and participates in melanosome transfer, which explains the pigmentary anomalies seen in DDD, and its mutation leads to abnormal proliferation of the pilosebaceous unit.^1^

Follicular DDD, first described by Singh et al. in 2013, is a distinct variant of Dowling-Degos disease characterized by punctate, folliculocentric hyperkeratotic and hyperpigmented papules, macules, pits, and comedo-like lesions.^1^ Lesions commonly affect the face, back, extremities, and flexures. Unlike classical DDD, follicular DDD lacks the typical reticulate pigmentation of flexures and shows histological changes confined to the follicular infundibulum, sparing the interfollicular epidermis.^2^

Dermatoscopy in follicular DDD reveals star-shaped or linear, thready pigmentation in a Chinese letter-like pattern, often centered around follicular openings, serving as a distinguishing feature of follicular DDD.^3^

In follicular DDD, histological changes are confined to the follicular infundibulum, showing features such as follicular plugging and horn cyst formation, while the interfollicular epidermis remains unaffected.^1^ Treatment options for Dowling-Degos disease include topical agents like retinoic acid and hydroquinone, as well as procedural interventions such as erbium-YAG laser and a combination of Q-switched Nd: YAG and fractional carbon dioxide lasers.

The differential diagnoses of follicular Dowling-Degos Disease (DDS) include classical dowling degos, Galli-Galli disease, familial dyskeratotic comedones, and comedonal Darier’s disease. Clinically, DDD begins as reticulate hyperpigmentation in intertriginous areas such as the axillae, groin, and inframammary folds, with gradual extension to sites like the trunk, inner thighs, upper arms, and face.^1^

Familial Dyskeratotic Comedones (FDC) is a rare autosomal dominant genodermatosis characterized by symmetrically distributed, hyperkeratotic, comedone-like papules.^4^ It typically begins around puberty, with lesions first appearing on the trunk or extremities and gradually spreading while sparing the palms, soles, and mucosal surfaces.^4^ FDC is suspected based on clinical resemblance to comedones, positive family history, and histological evidence of dyskeratosis. Lesions may also involve the face, scalp, and occasionally the genital area.^5^ Dermatoscopy has not been well described, but this report identified brownish follicular papules with central keratin plugs.^4^ Histopathology shows follicular invaginations filled with keratin-containing parakeratotic cells and melanin, along with dyskeratotic changes in deeper epithelial layers and occasional perivascular lymphocytic infiltration.^5^

Comedonal Darier’s disease is clinically distinct from the classic form due to its pronounced follicular involvement, leading to the development of large comedo-like lesions, primarily affecting the face and scalp, often accompanied by subtle features of classic Darier’s disease and poor response to therapy.^6^ The lesions are typically persistent and minimally symptomatic.

Clinically, it may present as either nodular lesions or multiple large comedo-like blackheads, particularly on the face, scalp, and upper trunk.^7^ Histopathologically, it demonstrates numerous dyskeratotic cells and occasional acantholytic cells confined to the follicular epithelium, along with dilated follicular ostia and distinguished from classic Darier’s disease by prominent follicular involvement and the presence of elongated dermal villi and papillary projections.^7^ Although usually minimally symptomatic and persistent, histopathology aids in diagnosis by revealing characteristic acantholytic and dyskeratotic cells within the follicular adnexal epithelium and dilated hair follicles.^6^

Follicular Dowling-Degos disease is a rare variant that can clinically mimic comedonal disorders, making diagnosis challenging. Dermoscopy and histopathology are essential in distinguishing it from its mimickers. Early recognition aids in accurate diagnosis, appropriate management, and genetic counseling.

## ORCID IDs

Anuja Yadav: 0000-0001-7392-4499

Meeta Singh: 0000-0003-2062-6628

Zoya Hasan: 0009-0004-5764-164X

## Financial support

This research did not receive any specific grant from funding agencies in the public, comercial, or not-for-profit sectors.

## Research data availability

Does not apply.

## Authors' contributions

**Sonika Garg:** Conceptualization, Methodology, Formal analysis, Investigation, Writing - original draft. **Anuja Yadav:** Conceptualization, Methodology, Formal analysis, Investigation, Writing - original draft. **Gajanand M. Antakanavar:** Conceptualization, Methodology, Formal analysis, Investigation, Writing - original draft. **Meeta Singh:** Conceptualization, Methodology, Formal analysis, Investigation, Writing - original draft. **Zoya Hasan:** Conceptualization, Methodology, Formal analysis, Investigation, Writing - original draft.

## Conflicts of interest

None declared.
